# Volatile Organic Compounds Induced upon Viral Infection in Cell Culture: Uniform Background Study with Use of Viruses from Different Families

**DOI:** 10.3390/molecules30234642

**Published:** 2025-12-03

**Authors:** Anna Karolina Matczuk, Julia Wolska, Maria Olszowy, Agata Kublicka, Adam Szumowski, Agata Kokocińska-Alexandre, Michał Dzięcioł, Jacek Łyczko, Martyna Woszczyło, Marcin J. Skwark, Antoni Szumny

**Affiliations:** 1 Department of Pathology, Division of Microbiology, The Faculty of Veterinary Medicine, Wrocław University of Environmental and Life Sciences, 50-375 Wrocław, Polandagata.kublicka@upwr.edu.pl (A.K.); 2 Department of Food Chemistry and Biocatalysis, Wrocław University of Environmental and Life Sciences, 50-357 Wrocław, Polandjacek.lyczko@upwr.edu.pl (J.Ł.); antoni.szumny@upwr.edu.pl (A.S.); 3 Institute of Biological Bases of Animal Production, Faculty of Animal Sciences and Bioeconomy, University of Life Sciences in Lublin, 20-950 Lublin, Poland; 4 Department of Reproduction and Clinic for Farm Animals, The Faculty of Veterinary Medicine, Wrocław University of Environmental and Life Sciences, 50-375 Wrocław, Poland; michal.dzieciol@upwr.edu.pl; 5 VetAI.bio Ltd., London UB9 5NJ, UKmarcin@skwark.pl (M.J.S.); 6 InstaDeep Ltd., London W2 1AS, UK

**Keywords:** virus infection, volatile organic compounds, VOC, equine viruses, EHV-1, EAV, ERBV, SPME, GC-MS, PCA

## Abstract

This study investigates the production of volatile organic compounds (VOCs) in RK-13 cells infected with three equine viruses representing different families: equine arteritis virus (EAV) (*Arteriviridae*), equine herpesvirus 1 (EHV-1) (*Herpesviridae*), and equine rhinitis B virus (ERBV) (*Picornaviridae*). VOCs, which are byproducts of cellular metabolism and potential non-invasive diagnostic markers, were analyzed using headspace solid-phase microextraction (HS-SPME) and gas chromatography–mass spectrometry (GC-MS). Since viruses do not possess intrinsic metabolic activity, the observed changes in the VOC profiles were attributed to host responses, such as metabolic reprogramming, oxidative stress, and apoptosis. We hypothesized that each viral infection induces distinct metabolic changes, resulting in characteristic VOC signatures that mirror the virus type, replication kinetics, and cytopathic effects. Notably, viruses with rapid cytopathic effects (e.g., EHV-1) were anticipated to trigger more pronounced VOC alterations. In our experimental design, RK-13 cells were infected at a multiplicity of infection of 1 and incubated for 24 h, 48 h, or 72 h. Distinct VOC profiles emerged, with significant elevations in compounds like 2-ethyl-1-hexanol, particularly in EHV-1 infections, and selective increases in acetophenone and benzaldehyde. Principal component analysis (PCA) of the VOC concentration data showed the clear separation of data from viruses from different families. These findings support the potential of VOC profiling as a rapid diagnostic tool for viral infections.

## 1. Introduction

Volatile organic compounds (VOCs) are chemicals that have a high vapor pressure at room temperature. Endogenous VOCs are products of metabolic activity in the body, and changes in these VOCs can be characteristic for age and specific disease [1]. Different biological samples, such as breath, urine, feces, body surface, and sweat, have been analyzed for their odor, serving as sources for detecting VOCs [2]. Various VOC types, including hydrocarbons (alkanes and alkenes), aldehydes, alcohols, ketones, aromatics, carboxylic acids, esters, ethers, and nitrogen- or sulfur-containing volatile compounds, have been identified in prior cancer research [3,4]. Also, the microbiome of an animal or human produces unique VOCs that can change under different circumstances, such as viral infection [5].

Viruses themselves do not possess any metabolic activity, and the composition of the viral particle (virion) is made up of lipids, DNA/RNA, and proteins, which are thought to be odorless; therefore, the production of VOCs is presumably associated with metabolic changes in infected cells associated with the viral replication cycle, oxidative stress, or induction of apoptosis [6]. In metabolomic studies, virus-infected cells are often characterized by increased aerobic glycolysis, elevated tricarboxylic acid cycle activity, enhanced oxidative phosphorylation, intensified glutaminolysis, and elevated fatty acid synthesis [7].

In the biomedical field, the analysis of volatile organic compounds (VOCs) produced by human fluids such as breath, urine, and feces, as well as tissues like skin and tumors, has been proposed as a non-invasive diagnostic tool for diseases [8,9,10].

Before the COVID-19 pandemic, there was very little work focused on the VOC analysis of viral infections [6,11,12,13]. In one study, VOC production by infected human B lymphocytes was analyzed for different subtypes of Influenza A. There were clear differences in the VOC profiles detected in noninfected and infected lymphocytes, as well as differences in compounds detected in samples derived from specific subtypes, times of infection, and multiplicities of infection (MOIs) [12]. In another study, the VOC analysis of cells infected with human respiratory viruses, including influenza and respiratory syncytial virus, was performed over time [13]. The study on the cell culture of rhinovirus and in volunteers infected with this virus showed that the decanes and other long-chain alkanes are increased in infected cells as well as in infected people, as measured in their breath [14]. Therefore, it is likely that the data generated from infected cell cultures can be translated into diagnostic tools for fast and rapid infectious disease diagnosis.

Although we have learned through many techniques which VOCs are produced during SARS-CoV-2 infection, both in studies performed on cell cultures as well as in affected people [15,16], little is known about the volatilomes of viruses of different families and, in particular, of animal viruses. As mentioned before, the volatilome of an infected human or animal is the resultant of many factors: the virus-induced metabolic changes in cells where it replicates, the VOCs produced upon immune stimulation, and the changes in the microbiome as an infection outcome [17]. However, preliminary in vitro studies on cell lines are fundamental for a proof of concept of the diagnostic, as well as for indication of potential VOC markers. As the microbiome of a particular individual is very variable, the immune response also differs across individuals, and the best markers come from infected cells themselves as a result of metabolome changes [18]. During acute infection (respiratory), most of the cells infected are epithelial cells. And these are the ones that produce characteristic VOC patterns. A recent meta-analysis on breath analysis in detection of COVID-19 through VOCs shows that many techniques used in detection of VOCs in patient breath showed high sensitivity and specificity (0.92 and 0.9, respectively) in detection of SARS-CoV-2 infection [19].

The development of new diagnostic techniques for acute viral infections would not have been possible without basic science and chemical analysis performed on virus-infected cell lines. Regardless of the extensive coronavirus research, there is still a need to look for differences in VOC profiles generated by infection of viruses from different families.

Volatile organic compounds (VOCs) generated during infections can be analyzed using various techniques, including gas chromatography–mass spectrometry (GC-MS) and gas chromatography–ion mobility spectrometry (GC-IMS). In this research, we utilized GC-MS in combination with solid-phase microextraction (SPME) for VOC analysis. Since its introduction by Arthur and Pawliszyn in 1990 [20], SPME has become a widely adopted method for VOC analysis, as it offers a straightforward and efficient approach by integrating sampling, isolation, and concentration into a single step [21,22]. During the process, heating the sample releases VOCs from the matrix, which then interact with the stationary phase. These compounds are subsequently separated in the GC column based on their chemical characteristics. Finally, the separated compounds are ionized and detected using a mass spectrometer [22].

Animal viruses are good models to study host–pathogen interactions, as most do not pose a threat to human personnel and results can be easily compared to human viruses. In this study we analyzed the VOC patterns of cells infected with equine arteritis virus (EAV), equine herpesvirus 1 (EHV-1), and equine rhinitis B virus (ERBV).

EAV is a virus in the *Nidovirales* order, *Arteriviridae* family, that is closely related to *Coronaviridae*. It is an enveloped RNA virus that replicates in cytoplasm, in replication centers of rearranged internal membranes. The virus causes a serious disease called equine viral arteritis that affects the respiratory and reproductive tracts. Upon viral infection, stallions may become persistent shedders of this virus [23].

Herpesviruses are a large family of enveloped viruses. They possess a large double-stranded DNA genome. The replication takes place in the nucleus, and the protein synthesis is divided into early gene expression and late gene expression. The EHV-1 is a causative agent of acute respiratory illness and abortion in pregnant mares, and the fatal myeloencephalopathy can also occur in horses [24].

Equine rhinitis B virus (ERBV) is a member of the family *Picornaviridae* and is a non-enveloped RNA virus that replicates in cytoplasm. After cellular rearrangements induced by the virus, the virions are released with the cell lysis and probably autophagy mechanisms [25,26]. ERBV causes acute respiratory disease in horses, and its incidence is rising worldwide [27].

The rabbit kidney 13 (RK-13) cell line is a continuous, epithelial cell line derived from the kidneys of a five-week-old rabbit. It is commonly used in the diagnosis of equine viruses, as recommended in manuals of the World Organisation for Animal Health, because they cause a visible cytopathic effect (CPE) in it, thereby enabling the isolation and identification of these pathogens [28,29].

The aim of this study is to analyze the volatilomes of three animal viruses that are able to replicate in one cell line in order to establish a uniform background. The hypothesis was that the metabolic changes induced upon viral infection vary in different virus species, resulting in the production of specific VOCs or changes in the concentration of VOCs already produced in the cells. A potential VOC marker for equine respiratory disease might be developed in the future.

## 2. Results

**Figure 1:**
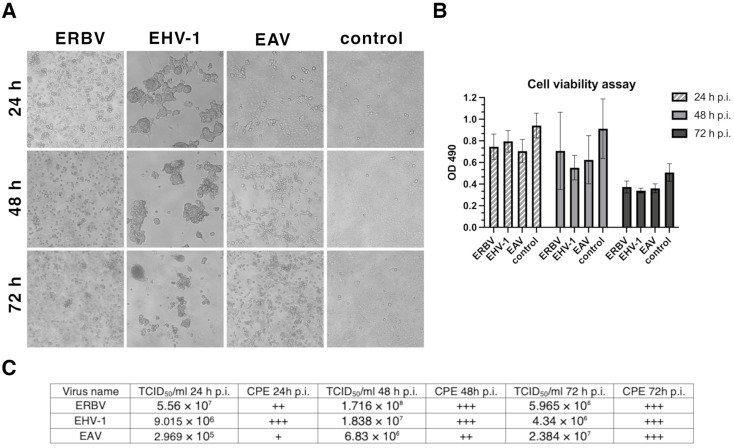
(**A**) Bright-field images of RK-13 cell monolayers: uninfected control and cultures infected with EHV-1 (equine herpesvirus 1), EAV (equine arteritis virus), or ERBV (equine rhinitis B virus). (**B**) Cell viability measured by MTS assay (OD, optical density). (**C**) Virus titers determined by TCID50 assay and cytopathic effect (CPE) scoring (“+” mild; “++” moderate; “+++” extensive). p.i., post-infection.

The CPE of RK-13 cells infected with EHV-1, EAV, or ERBV was visualized daily, before GC-MS sample collection. The CPE after virus infection was the most pronounced for EHV-1 at 24 h p.i., followed by ERBV (Figure 1A). The cytotoxicity assay showed that the cells were viable 24 h after infection, despite the visible CPE, but the viability decreased slightly after 48 h, while only half of the cells remained viable after 72 h, even in control cells (Figure 1B).

The CPE caused by EAV increased steadily over time, reaching a maximum at 72 h p.i. The titers of the viruses were the highest at 48 h p.i. for EHV-1 and ERBV, while for EAV, the highest titer was reached at 72 h p.i. (Figure 1C).

**Figure 2:**
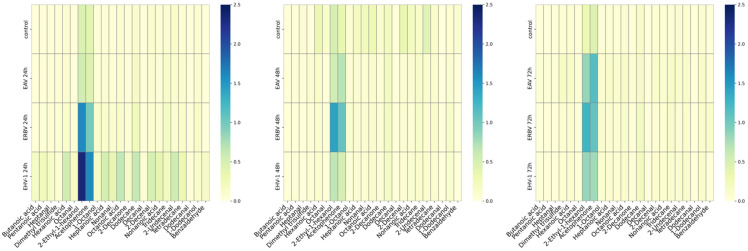
Heatmaps of mean concentrations of volatile organic compounds (VOCs) in infected cultures at different timepoints after infection (p.i., post-infection): 24 h p.i. (**left panel**); 48 h p.i. (**middle panel**); 72 h p.i. (**right panel**). Control—uninfected cells; EHV-1—equine herpesvirus 1; EAV:—equine arteritis virus; ERBV—equine rhinitis B virus. Scales in μg per g.

In the cell culture medium (background), we identified 22 compounds, while in cells, we identified 23 compounds, regardless of the infection state. Dimethyl trisulfide was the only absent VOC in the medium (Table 1). Other compounds were detected in the samples as well in the background medium, but in different amounts. Different classes of organic compounds, i.e., alcohols: 2-ethyl 1-hexanol, 1-octanol, and 1-dodecanol; ketones: acetophenone and 2-decanone; aldehydes: heptanal, octanal, nonanal, decanal, dodecanal, benzaldehyde, 2-decenal, and 2-undecenal; alkanes: dodecane, tridecane, and tetradecane; carboxylic acids: butanoic acids, pentanoic acid, hexanoic acid, heptanoic acid, octanoic acid, and nonanoic acid. As mentioned above, the dimethyl trisulfide (a sulfur-containing compound) was identified only in samples with cells (infected or uninfected cells), but not in the cell culture medium itself. The highest concentrations were observed for 2-ethyl-1-hexanol and acetophenone, especially for the infected cells (Figure 2).

**Figure 3:**
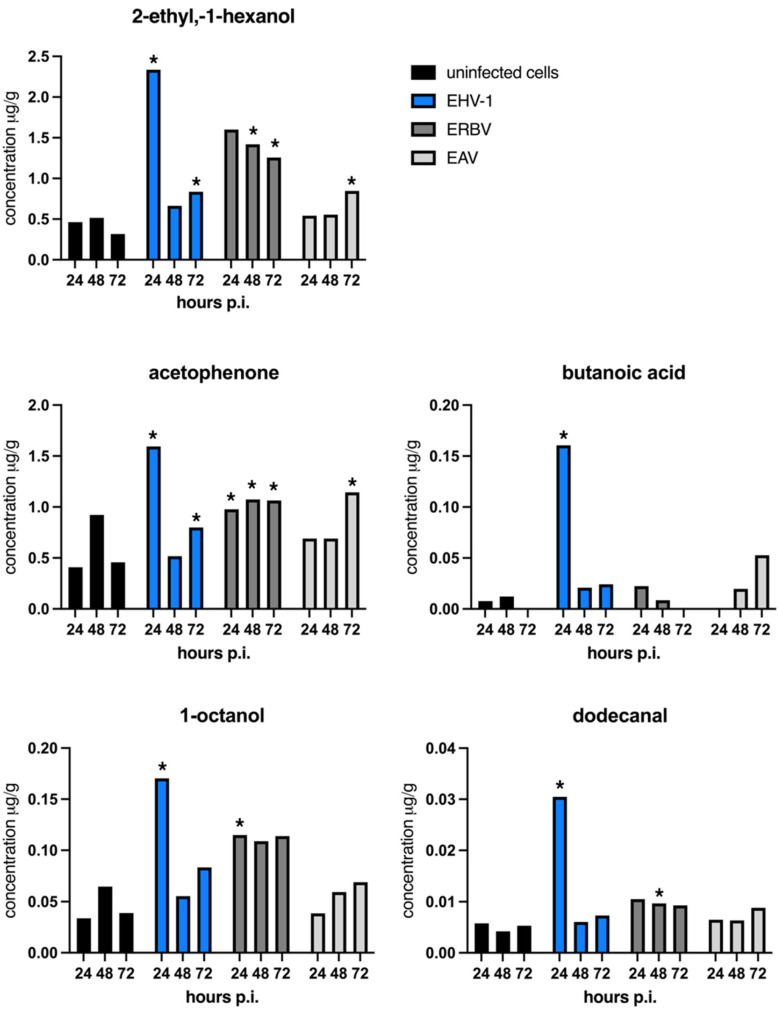
Mean concentrations of individual VOCs (μg per g) across experimental timepoints. A star (*) denotes a statistically important increase in the concentration of a particular VOC in virus-infected samples in comparison to the concentration in uninfected control samples at an indicated timepoint; *p* < 0.05 (one-way ANOVA). p.i.—post-infection; EHV-1—equine herpesvirus 1; ERBV—equine rhinitis virus B; EAV—equine arteritis virus. Graphs were generated with GraphPad Prims.

At 24 h post-infection, the highest concentrations were observed for 2-ethyl-1-hexanol and acetophenone. However, the increase in 2-ethyl-1-hexanol was statistically significant only in EHV-1-infected cells (herpesvirus), while acetophenone was significantly elevated in EHV-1- and ERBV-infected cells. 1-octanol was significantly increased in EHV-1 and ERBV samples, while butanoic acid was significantly increased in herpesvirus-infected cells (Figure 3).

At 48 h post-infection, the only statistically significant changes were seen for 2-ethyl-1-hexanol, acetophenone, and dodecanal in ERBV-infected cells, but not in EAV or EHV-1 infections (Figure 3).

At 72 h post-infection, the significant elevation of 2-ethyl-1-hexanol was observed across all virus-infected cells. Acetophenone remained significantly increased—with the highest levels seen in the RNA viruses (EAV and ERBV) and a more modest rise in EHV-1. Benzaldehyde emerged as a new marker, significantly elevated in EAV but not significantly elevated in ERBV infections. This compound was not detected in EHV-1 samples or uninfected controls (Supplementary Table S1).

An analysis of variance revealed that at 24 h post-infection, the sample group (virus vs. control) had a significant effect on the VOC profiles (F(15, 22.49) = 2.81, *p* = 0.013, partial η^2^ = 0.652), with an observed noncentrality parameter of 42.11 and observed power of 0.934, indicating that the VOC compositions differed significantly between virus-infected and control samples even at this early stage. At 48 h post-infection, the effect of the virus type on VOCs approached significance (F(36, 3.68) = 3.64, *p* = 0.121, partial η^2^ = 0.973), with an observed noncentrality parameter of 131.21 and observed power of 0.437, reflecting a very large effect size. By 72 h post-infection, a significant overall effect of the virus type on the combined VOC profiles was observed (F(30, 3.61) = 7.95, *p* = 0.036, partial η^2^ = 0.985, observed power = 0.75), demonstrating that virus infection substantially altered the VOC composition compared with controls, with a very large effect size.

EHV-1 infection, which exhibited the fastest and most pronounced cytopathic effects as well as the highest titer at 24 h post-infection, showed the most abundant and significant changes in VOC production, including acetophenone, 2-ethyl-1-hexanol, butanoic acid, and 1-octanol. Similar significant increases in these compounds were noted in ERBV-infected cells, whereas in EAV-infected cells, only 2-ethyl-1-hexanol was significantly elevated at 24 h. This compound remained significantly altered in ERBV at the 48 h timepoint. Notably, acetophenone also increased significantly in the control sample at 48 h, suggesting a potential non-specific, distress-related response rather than a virus-specific effect. Furthermore, although dodecanal was detected in small amounts at 48 h, its increase was statistically significant in all virus-infected cells, especially in ERBV (an unenveloped virus). Finally, at 72 h, benzaldehyde was significantly elevated only in RNA virus infections (EAV and ERBV), while both acetophenone and 2-ethyl-1-hexanol reached their highest statistical significance in ERBV and EAV infections, with less pronounced changes in EHV-1.

Other compounds showed increased levels as well, especially in EHV-1 samples at the 24 h timepoint and in EAV at the 72 h timepoint; however, these changes were not statistically significant.

**Figure 4:**
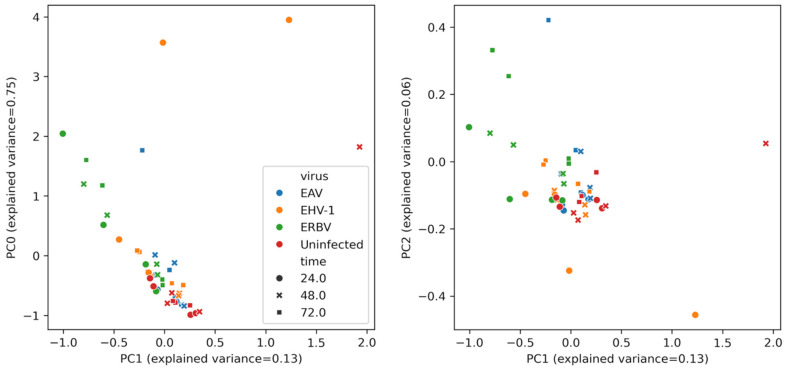
Multivariate analysis of VOC profiles in virus-infected and uninfected cell culture samples. Principal component analysis (PCA) of VOC concentration data, showing separation of EAV-infected (blue), ERBV-infected (green), EHV-1-infected (orange), and control uninfected (red) samples along the first principal component and separation time-wise along the second principal component. Data shown for both biological replicates. The legend for both plots is shared.

A multivariate analysis of the concentrations of the compounds (after removing background, i.e., concentrations of compounds in the medium) is presented in Figure 4. Uninfected samples are clustered on the left, whereas infected samples lay on the manifold below and to the left in the PC0-PC1 plot. We observe that the EHV-1 points (orange) tend to have high values along PC0, while the ERBV points are located to the left of the PC1 axis. The points of EAV, a virus that produced a less pronounced CPE, tend to be relatively low along the PC0 axis. The gradient structure of the data in the PC0-PC1 view indicates a strong statistical coupling between these principal component vectors.

## 3. Discussion

In RK-13 cells, the timing and severity of the CPEs and peak virus titers followed the known biology of each virus. EHV-1 produced the earliest and most conspicuous CPEs—syncytia, cell rounding, and detachment—by 24 h p.i., consistent with herpesviral membrane fusion activity and the extensive, stage-wise rewiring of the host cytoskeleton and gene expression that accompanies the early, intermediate, and late phases of the herpesviral lytic cycle. ERBV showed CPEs the second-fastest, in line with picornaviruses’ short replication cycles (often ≤6–8 h) and non-enveloped egress that proceeds primarily by cell lysis (and, in some systems, autophagy-linked pathways) [31,32]. By contrast, EAV displayed a gradual, “milder” CPE that accumulated through 72 h p.i., consistent with arteriviral replication on virus-induced double-membrane vesicles, the extensive remodeling of intracellular membranes, and particle release via the secretory pathway rather than abrupt cell lysis [33,34]. Cell viability measurements mirrored these trends: despite clear CPEs for EHV-1 and ERBV, their viability remained high at 24 h and declined modestly at 48 h, with a larger drop by 72 h. The 72 h decline seen even in controls likely reflects confluence and medium exhaustion rather than virus-specific toxicity. Virus production peaked at 48 h for EHV-1 and ERBV, whereas EAV reached its maximum at 72 h, indicating that a visible CPE does not strictly predict peak infectivity, and that EAV’s slower membrane-associated biogenesis shifts yield to later timepoints [34,35]. Overall, the kinetics of the CPE, viability, and titer in our system are in agreement with the established replication strategies of herpes-, picorna-, and arteriviruses in cell culture.

Our results indicate that 2-ethyl-1-hexanol is closely related to CPEs and viral titers, as it was elevated at various timepoints across all virus species, which matched these observations. This observation suggests that its increase may represent a general marker of cellular stress generated due to viral infection. Similar findings were reported in studies on human breast cancer and lung cancer cell cultures, where 2-ethyl-1-hexanol, indicative of cellular stress, and cyclohexanol were among the major compounds identified [4]. Moreover, 2-ethyl-1-hexanol has been detected in RSV-infected cells, but not in those infected with influenza [13]. In our study, the correlation of 2-ethyl-1-hexanol with both cytotoxicity and viral yield indicates that it may be produced as a byproduct of augmented metabolism—likely stemming from lipid metabolism or fatty acid oxidation—even though its precise biosynthetic pathway in mammalian cells remains unclear. Although this compound can also derive from the degradation of plasticizers such as di(2-ethylhexyl) phthalate (DEHP), our controls using medium incubated in plastic cell culture bottles (with background extraction) strongly support an endogenous origin, particularly in light of the oxidative stress induced by viral infection [36,37].

Acetophenone was another VOC detected in our experiments that is generated endogenously, potentially through the degradation of aromatic amino acids like phenylalanine or via other enzymatic reactions. It is considered a marker of altered metabolic states or cellular stress. For example, breath analyses have reported higher acetophenone levels in healthy individuals compared to those with chronic liver disease [38]. Additionally, studies on Zika-infected mice showed that infected animals emitted approximately tenfold higher levels of acetophenone than uninfected controls, and this compound was implicated in attracting mosquitoes [30]. In our in vitro model, where we ensured sterility (negative mycoplasma tests, use of antibiotics, and aseptic handling), the presence of acetophenone in both infected and control RK-13 cells suggests that it may indeed be a VOC product of the cells themselves.

Significant differences among groups were also observed for 1-octanol and butanoic acid in EHV-1-infected cells at 24 h post-infection. These compounds may arise from altered lipid metabolism, a known effect of DNA viruses—especially herpesviruses—which can induce changes in lipid biosynthesis and regulation [39]. At 48 h post-infection, a statistically significant increase in dodecanal levels was observed in ERBV-infected cells. This likely reflects the breakdown of membrane lipids due to cellular damage, consistent with ERBV’s reliance on cell lysis for viral release [40]. In contrast, EAV infection, which does not induce cell lysis but rather causes cell rounding and cytoskeletal rearrangements with virion budding via the secretory pathway [33], showed delayed VOC changes, with significant increases in 2-ethyl-1-hexanol and acetophenone observed only at later timepoints.

During the COVID-19 pandemic, breath analyses in SARS-CoV-2-infected patients commonly detected acetaldehyde, nonanal, octanal, and heptanal [19]. In our study, although nonanal, octanal, and heptanal were occasionally elevated, these differences were not statistically significant compared to controls. This may be attributed to the cell culture model used here, which lacks the in vivo immune response that can further modulate VOC release during infection.

We employed an MOI of 1 to ensure uniform infection and significant metabolic changes. Consistent with previous reports [11], our data indicate that ERBV and EHV-1 induce robust VOC changes by 24 h, whereas EAV-induced alterations become more apparent at 48 and 72 h. The differences in timing likely reflect variations in the replication cycles and pathogenic mechanisms of these viruses mentioned earlier. It is noteworthy that most VOCs were also detected in the culture medium, likely due to the presence of fetal bovine serum—a common supplement of animal origin that may itself contribute VOCs. However, the absence of dimethyl trisulfide in the medium, a product of sulfur-containing amino acid metabolism (e.g., cysteine and methionine) that we identified as a marker of cells, further supports the cellular origin of the detected VOCs.

Viral infections often induce a metabolic shift toward aerobic glycolysis (the Warburg effect) and favor fatty acid synthesis over fatty acid oxidation [41,42]. This shift can lead to enhanced lipid peroxidation and the formation of reactive aldehydes (such as hexanal, nonanal, and heptanal) and ketones (such as acetone and 2-butanone). The induction of VOCs like 2-ethyl-1-hexanol reflects these alterations in lipid handling and membrane turnover. Furthermore, 1-octanol, likely generated through lipid peroxidation, underscores the impact of oxidative stress on VOC production during viral infection.

Principal component analysis (PCA) of the volatilome reduces the dimensionality of complex VOC datasets and reveals the multivariate structure that single-compound tests may miss. It has been widely applied—from food quality and plant defense to environmental monitoring and non-invasive diagnostics from breath or urine—to discriminate samples by odor fingerprints rather than by individual markers [43]. In microbiology, PCA of VOC profiles can robustly separate bacterial species such as *Escherichia coli*, *Staphylococcus aureus*, and *Pseudomonas aeruginosa*, even across different culture media, highlighting the stability of multivariate odor signatures [44]. Related work in bioprocessing shows that VOC emissions from cultured cells correlate with cell expansion in bioreactors (e.g., CHO and T-cell systems), and PCA on such signals has been used to monitor confluency and growth dynamics [45].

In this context, our cell culture data extend volatilome PCA to virology. Despite relatively few statistically significant univariate differences among individual VOCs, the PCA revealed clear, well-defined separation both by time post-infection and by virus, indicating that distinct viral species can be discriminated by their composite VOC signatures without relying on a single specific marker.

To the best of our knowledge, this is the first demonstration of the PCA-based discrimination of virus-derived volatilomes in a controlled cell culture model. Beyond classification, the temporal trajectories in PCA space suggest that volatilome readouts could inform operational decisions—such as pinpointing the optimal harvest time in vaccine production—by providing a non-invasive proxy for infection progression.

Collectively, these findings underscore the potential of VOC profiling in vitro as a diagnostic and process analytical tool and offer insight into the distinct metabolic perturbations elicited by different viral pathogens.

## 4. Summary

In summary, dimethyl trisulfide was identified as a marker of cellular metabolism since it was absent in the cell culture medium but consistently present in samples containing cells. In contrast, 2-ethyl-1-hexanol and acetophenone emerged as clear indicators of viral infection, with their concentrations correlating with viral replication, cytopathic effects, and changes over the course of infection. Butanoic acid was significantly elevated in infections caused by enveloped viruses (EHV-1 and EAV) but not in those caused by the unenveloped ERBV, suggesting increased fatty acid and membrane lipid metabolism in enveloped virus infections. Although no virus-specific VOCs were observed under our experimental conditions, the strength of this study lies in its simultaneous evaluation of viruses from distantly related families. Nonetheless, the subtle differences in metabolic responses across virus families and similar metabolic alterations pose challenges for VOC-based viral detection.

## 5. Study Limitations

Several limitations should be acknowledged. First, the RK-13 cell line, while offering a uniform and reproducible model, represents an immortalized system that may not fully capture the metabolic complexity of primary cells. This could influence both the range and concentration of VOCs detected. Moreover, RK-13 cells are routinely used in various diagnostic assays beyond equine virus studies, and their inherent metabolic characteristics may confound responses specific to equine viral infections. Finally, the sensitivity and specificity of the analytical technique (GC-MS) and potential variability in fiber absorption may have resulted in certain VOCs falling below the detection limit or being underestimated. These limitations underscore the need for future studies employing additional cell models, more sensitive analytical methods, and in vivo approaches to validate and extend these findings.

## 6. Materials and Methods

### 6.1. Cell Lines

The stable cell line rabbit kidney, RK-13 cells (ATCC CCL-37), was grown and maintained in EMEM (Capricorn Scientific, Ebsdorfergrund, Germany) supplemented with 10% fetal calf serum (FCS) sourced from Biological Industries (Cromwell, CT, USA), 100 U/mL of penicillin, 100 µg/mL of streptomycin, 1% L-glutamine, and 1% non-essential amino acid solution (Sartorius, Goettingen, Germany). The cells were maintained in a controlled environment at 37 °C with 5% CO_2_ and 95% humidity throughout the experiments.

### 6.2. Viruses

Equine arteritis virus (EAV), the Bucyrus reference strain, equine herpesvirus 1, the Valencia strain [36], and equine rhinitis B virus (ERBV), an Australian reference strain 1331/3/96, were propagated in RK-13 once and stored at −80 °C.

### 6.3. Titration

TCID 50 was performed on the RK-13 cell line for titration of the cell culture supernatants of infected and mock cells collected at the three indicated timepoints.

### 6.4. Cell Viability Assay

Cell viability was evaluated with a 3-(4,5-dimethylthiazol-2-yl)-5-(3-carboxymethoxyphenyl)-2-(4-sulfophenyl)-2H-tetrazolium (MTS) assay kit according to the manufacturer’s instructions (Abcam, Cambridge, UK) on a 96-well plate in triplicate 24, 48, and 72 h p.i. The absorbance was read in the Epoch microplate spectrophotometer (Agilent, BioTek, Warsaw, Poland) at 490 nm.

### 6.5. GC-MS Sample Preparation

The three T75 bottles of RK-13 cells were infected with MOI 1 of viruses: EAV, EHV-1, ERBV, or mock infection. Infected cells were maintained in EMEM supplemented with 0.5% FBS, 1% L-glutamine, 100 U/mL of penicillin, and 100 µg/mL of streptomycin. The control uninfected cells were prepared in the same manner as the infected ones (which included “infection” with medium, washing steps with PBS, and medium exchange). The background control was prepared in parallel by placing the medium in bottles (without the cells). The background sample was prepared in order to specify the exogenous VOCs from the medium, FBS, and plastic bottles. The bright-field pictures of cells were taken with an Axio Observer 1 inverted microscope (Zeiss, Oberkochen, Germany) equipped with a 10× objective.

Cells/control medium were incubated for 24 h, 48 h, or 72 h, an aliquot of 200 µL of cell culture supernatant was taken for titer estimation, and then the cells were scraped of the surface together with the medium and transferred to 10 mL chromatography glass vials (2 for each timepoint and virus for technical repetition) and stored at −80 °C until further processing. The experiment was performed in biological duplicate, in two technical replicates and two analytical replicates.

### 6.6. Headspace Solid-Phase Microextraction (HS-SPME)

Before analysis, 2-undecanone (99% purity) (Sigma-Aldrich, Steinheim, Germany) was added to the frozen matrix. For the extraction of volatile compounds, the following was used: SPME Arrow 1.10 mm 20 mm length, DVB/C-WR/PDMS (CTC Analytics AG, Zwingen, Switzerland). Before running each sample, the fiber was conditioned at 250 °C for 2 min and 3 min after desorption. Sample vials were incubated and extracted on a PAL autosampler in designated modules. Sample vials were incubated at 70 °C for 15 min, extracted at 70 °C for 30 min, and desorbed in split 1 mode at 250 °C for 3 min; the helium (analytical purity: 99.99997%, Air Products, Warsaw, Poland) flow rate was 1 mL/min.

### 6.7. Determination of VOCs

The analysis of VOCs on the SPME fiber was performed with a Shimadzu GCMS-QP2020 (Shimadzu Company, Kyoto, Japan) apparatus (single quadrupole). The separation was performed on a capillary column, Zebron ZB-5 (30 m × 0.25 mm × 0.25 µm) (Phenomenex, Torrance, CA, USA). The oven temperature was kept at 50 °C for 3 min, and it was heated up to 150 °C at a rate of 5 °C min^−1^, reaching a final temperature of 250 °C at 15 °C, and it was held at this temperature for 5 min. The total run time was 34.67 min. The MS parameters included electron ionization at 70 eV, an interface temperature of 250 °C, an ion source temperature of 250 °C, and Full Scan mode. Analysis was performed between 1 and 30 min with a range from 40 *m*/*z* to 400 *m*/*z*.

Identification of VOCs was carried out by comparing the experimentally obtained mass spectra and calculated linear retention indices (LRIs) with reference data. The LRIs were determined using a standard mixture of n-alkanes (C7–C30; Sigma-Aldrich, Steinheim, Germany) run under identical chromatographic conditions. Mass spectral data were interpreted using automated and manual matching against multiple spectral libraries, including the NIST WebBook, NIST23 databases. Where applicable, additional verification was performed through comparison with published literature data. Compounds were considered to be tentatively identified when both the mass spectrum match and LRI deviation fell within accepted confidence thresholds. This integrative approach enabled the reliable annotation of VOCs present in the headspaces of both virus-infected and uninfected cell cultures. Before analysis, samples were checked for the absence of 2-undecanone, which was used as an internal standard.

Compound concentrations were determined using the internal standard (IS) method. For each sample, peak areas of the analytes were extracted from the GC-MS chromatograms and normalized by the peak area of the internal standard to correct for ations in the injection volume and instrument response. The normalized ratios were then converted to absolute concentrations in µg per g of sample by multiplying by the known amount of internal standard added and dividing by the sample mass.

### 6.8. Statistical Analysis

Statistical analyses, in terms of analysis of variance (ANOVA) and multivariate analysis (heatmap matrices), were performed using Statistica version 13.1 (StatSoft, Tulsa, OK, USA). For each timepoint (24, 48, 72 h post-infection), a one-way ANOVA followed by Tukey’s HSD test was performed to compare VOC concentrations between virus-infected and control noninfected samples.

Data visualization in the form of a heatmap was performed in Python using the pandas, seaborn, and matplotlib libraries. Input data in TSV format were imported and processed with pandas, which enabled the correct handling of the decimal separator and the separation of the numerical part of the data matrix from the sample-identifying column. For visualization, the seaborn library was employed. To ensure clarity of presentation, automatic layout adjustment was applied, and the figure size was adapted to accommodate a larger number of variables.

GraphPad Prism version 10.4.1 was used to generate graphs (Figure 1B and Figure 3).

Statistical analysis of chromatography data for principal component analysis (PCA) was performed using tailor-made Python code, leveraging the pandas library for data handling, SciPy for statistical analysis, as well as the scikit-learn library for the training and evaluation of the machine learning models.

## Figures and Tables

**Table 1:** The table summarizes all VOCs identified in this study, including their experimental linear retention indices (LRIs), corresponding literature LRIs, retention index deviations (ΔLRIs), mass spectral match factors, characteristic fragment ions (*m*/*z*), and relevant comments on biological relevance or infection-associated changes. Experimental LRIs were calculated using an n-alkane series and compared with published reference values. VOCs showing statistically significant elevation at specific timepoints post-infection (p.i.) are indicated accordingly. Literature comments include previously reported associations with viral infections (e.g., SARS-CoV-2, RSV, Zika virus) or other biological systems where applicable (in green font). The internal standard (2-undecanone) is included for reference.

No.	VOC Name	Experimental LRI	Literature LRI	ΔLRI	MS Match (%)	Key Ions (*m*/*z*)	CAS	Molecular Formula	Comments
1.	Butanoic acid	805	803	−2	90	60, 73, 88	107-92-6	C_4_H_8_O_2_	Significantly elevated 24 h p.i. with EHV-1.
2.	Pentanoic acid	897	901	4	91	60, 73, 87	109-52-4	C_5_H_10_O_2_	
3.	Heptanal	902	901	−1	81	55, 70	111-71-7	C_7_H_14_O	Elevated in SARS-CoV-2 studies. [19]
4.	Benzaldehyde	967	962	−5	95	77, 105, 106	100-52-7	C_7_H_6_O	Significantly elevated 72 h p.i. with EAV.
5.	Dimethyl trisulfide	975	971	−4	85	60, 126	3658-80-8	C_2_H_6_S_3_	Absent in medium.
6.	Hexanoic acid	987	990	3	95	60, 101, 115	142-62-1	C_6_H_12_O_2_	
7.	Octanal	1004	1003	−1	86	70, 110	124-13-0	C_8_H_16_O	Elevated in SARS-CoV-2 studies [19]
8.	2-ethyl-1-hexanol	1032	1030	−2	95	70, 157	104-76-7	C_8_H_18_O	Elevated in infected cells in this study. Reported in cancer research studies. [4] RSV-infected cells. [13]
9.	Acetophenone	1071	1066	−5	97	77, 105	98-86-2	C_8_H_8_O	Elevated in infected cells in this study. Elevated in Zika virus-infected mice [30]
10.	1-Octanol	1075	1070	−5	92	55, 70, 130	111-87-5	C_8_H_18_O	Significantly elevated 24 h p.i. with EHV-1 and ERBV.
11.	Heptanoic acid	1079	1078	−1	89	87, 101, 129	111-14-8	C_7_H_14_O_2_	
12.	Nonanal	1106	1104	−2	96	57, 70, 98	124-19-6	C_9_H_18_O	Elevated in SARS-CoV-2 studies. [19]
13.	Octanoic acid	1176	1180	4	82	101, 115, 143	124-07-2	C_8_H_16_O_2_	
14.	2-Decanone	1194	1193	−1	87	71, 170	693-54-9	C_10_H_20_O	
15.	Dodecane	1199	1200	1	96	57, 71, 170	112-40-3	C_12_H_26_	
16.	Decanal	1206	1206	0	96	70, 95, 155	112-31-2	C_10_H_20_O	
17.	2-Decenal	1263	1263	0	92	69, 83, 153	3913-71-1	C_10_H_18_O	
18.	Nonanoic acid	1268	1273	5	94	87, 101, 129	112-05-0	C_9_H_18_O_2_	
19.	2-Undecanone	1294	1294	0	96	43, 58, 71	112-12-9	C_11_H_22_O	Internal standard
20.	Tridecane	1298	1300	2	96	57, 71, 85	629-50-5	C_13_H_28_	
21.	2-Undecenal	1364	1367	3	86	83, 124	2463-77-6	C_11_H_20_O	
22.	Tetradecane	1397	1400	3	97	83, 113	629-59-4	C_14_H_30_	
23.	Dodecanal	1408	1409	1	93	57, 70, 156	112-54-9	C_12_H_24_O	Significantly elevated 24 h p.i. in EHV-1 and 48 h p.i. with ERBV.
24.	1-Dodecanol	1476	1474	−2	92	55, 70, 168	112-53-8	C_12_H_26_O	

## Data Availability

Raw data from GC-MS analysis were deposited in an open-access repository: DOI:10.57755/gcar-y540.

## Supplementary Materials

The following supporting information can be downloaded at https://www.mdpi.com/article/10.3390/molecules30234642/s1, Table S1: Concentrations of VOCs obtained in each readout.

## Abbreviations

The following abbreviations are used in this manuscript:

VOC	Volatile organic compound
CPE	Cytopathic effect
EHV-1	Equine herpesvirus 1
EAV	Equine arteritis virus
ERBV	Equine rhinitis B virus
TCID50	Tissue culture infectious dose 50
MOI	Multiplicity of infection
RK-13	Rabbit kidney 13
GC-MS	Gas chromatography–mass spectrometry
GC-IMS	Gas chromatography–ion mobility spectrometry
PCA	Principal component analysis
