# Supplementary material for: Volatile Organic Compounds Induced upon Viral Infection in Cell Culture: Uniform Background Study with Use of Viruses from Different Families
Source: Molecules. 2025 Dec 3;30(23):4642. doi: 10.3390/molecules30234642 (PMC12692951; doi:10.3390/molecules30234642)
Supplement: Supplementary file 1 [file molecules-3972259-supplementary.pdf › molecules-3972259-supplementary.pdf]

RT.	Sample	medium	medium	control 2	control 2	control 2	control 2	control 4	control 4	control 4	control 4	control 7	control 7	control 7
5.085	Butanoic acid	0.02	0.037	0	0.031	0	0	0.049	0	0	0	0	0	0
7.135	Pentanoic acid	0.027	0.049	0.038	0.043	0	0	0.055	0	0	0	0	0	0
7.325	Heptanal	0.027	0.029	0.026	0.021	0.016	0.014	0.025	0.023	0.028	0.225	0.022	0.021	0.02
9.425	Benzaldehyde	1.375	1.679	0	0	0	0	0	0	0	0.305	0	0	0
9.690	Dimethyl trisulfide	0	0	0.041	0.025	0	0	0	0	0	0	0.077	0.077	0.021
10.085	Hexanoic acid	0.044	0.096	0.064	0.074	0.119	0.049	0.117	0.065	0.091	0.907	0.071	0.069	0.05
10.665	Octanal	0.024	0.023	0.025	0.02	0.016	0.016	0.104	0.015	0.027	0.255	0.021	0.019	0.023
11.465	1-hexanol, 2-ethyl- /2-Propyl-1-pentanol	0.461	0.557	0.827	0.739	0.147	0.145	0.535	0.454	0.128	0.944	0.407	0.364	0.18
12.695	Acetophenone	0.846	1.007	0.53	0.428	0.339	0.341	0.374	0.28	0.381	2.653	0.386	0.413	0.575
12.820	1-Octanol	0.028	0.036	0.05	0.051	0.018	0.016	0.064	0.042	0.024	0.129	0.054	0.046	0.017
12.965	Heptanoic acid	0.042	0.08	0.066	0.063	0.05	0.04	0.111	0.06	0.058	0.59	0.069	0.065	0.045
13.850	Nonanal	0.064	0.07	0.049	0.045	0.058	0.058	0.058	0.038	0.111	0.784	0.044	0.047	0.057
15.940	Octanoic acid	0.059	0.105	0.09	0.086	0.077	0.06	0.126	0.077	0.083	0.865	0.087	0.094	0.072
16.515	2-Decanone	0.031	0.035	0.039	0.035	0.028	0.034	0.033	0.038	0.042	0.256	0.035	0.038	0.034
16.695	Dodecane	0.175	0.187	0.174	0.133	0.043	0.052	0.213	0.153	0.059	0.451	0.199	0.149	0.101
16.890	Decanal	0.017	0.014	0.008	0.005	0.009	0.009	0.009	0.008	0.011	0.089	0.008	0.008	0.009
18.480	2-Decenal	0.051	0.059	0.054	0.043	0.154	0.103	0.07	0.054	0.152	1.104	0.055	0.046	0.086
18.610	Nonanoic acid	0.037	0.065	0.046	0.051	0.055	0.047	0.082	0.039	0.062	0.632	0.051	0.055	0.045
19.535	Tridecane	0.123	0.111	0.224	0.107	0.02	0.018	0.2	0.132	0.02	0.153	0.076	0.05	0.032
21.270	2-Undecenal	0.043	0.057	0.059	0.049	0.179	0.151	0.076	0.05	0.205	1.315	0.055	0.055	0.108
22.210	Tetradecane	0.088	0.08	0.061	0.063	0.045	0.046	0.088	0.057	0.043	0.224	0.064	0.062	0.063
22.460	Dodecanal	0.026	0.021	0.005	0.005	0.007	0.006	0.007	0.004	0.006	0	0.004	0.005	0.007
23.935	1-Dodecanol	0.067	0.078	0.071	0.071	0.076	0.048	0.104	0.067	0.073	0	0.082	0.077	0.077

RT.	Sample	EAV 24h	EAV 24h	EAV 24h	EAV 24h	EAV 48h	EAV 48h	EAV 48h	EAV 48h	EAV 72h	EAV 72h	EAV 72h	EAV 72h	ERBV 24h	ERBV 24h	ERBV 24h	ERBV 24h
5.085	Butanoic acid	0	0	0	0	0.058	0	0	0.021	0.054	0.105	0.022	0.03	0	0.09	0	0
7.135	Pentanoic acid	0.053	0.043	0	0	0.075	0.085	0.034	0.028	0.061	0.142	0.027	0	0.055	0.107	0	0.04
7.325	Heptanal	0.029	0.029	0.022	0.02	0.038	0.051	0.025	0.016	0.036	0.095	0.036	0.032	0.036	0.053	0.02	0.025
9.425	Benzaldehyde	0	0	0	0	0	0.048	0	0	0.032	0.086	0	0	0	0	0	0
9.690	Dimethyl trisulfide	0.089	0.047	0	0	0.143	0.134	0.02	0.022	0.152	0.416	0.023	0.018	0.068	0.131	0	0
10.085	Hexanoic acid	0.1	0.076	0.061	0.065	0.132	0.153	0.084	0.077	0.117	0.315	0.04	0.065	0.092	0.173	0.053	0.121
10.665	Octanal	0.027	0.021	0.019	0.015	0.033	0.036	0.019	0.018	0.031	0.082	0.02	0.018	0.037	0.052	0.013	0.019
11.465	1-hexanol, 2-ethyl- /2-Propyl-1-pentanol	0.81	0.669	0.308	0.384	0.971	0.713	0.278	0.258	0.675	1.956	0.434	0.322	1.771	2.921	0.671	1.045
12.695	Acetophenone		0.383	0.407	0.44	0.916	0.96	0.413	0.471	0.901	2.562	0.49	0.625	0.853	1.966	0.427	0.661
12.820	1-Octanol	0.065	0.039	0.026	0.025	0.086	0.095	0.03	0.027	0.045	0.166	0.035	0.029	0.161	0.206	0.032	0.061
12.965	Heptanoic acid	0.095	0.067	0.054	0.064	0.103	0.13	0.073	0.069	0.1	0.254	0.068	0.082	0.073	0.158	0.06	0.102
13.850	Nonanal	0.063	0.048	0.052	0.038	0.073	0.093	0.053	0.05	0.067	0.172	0.056	0.048	0.098	0.141	0.029	0.054
15.940	Octanoic acid	0.11	0.085	0.098	0.085	0.124	0.155	0.095	0.088	0.112	0.274	0.092	0.098	0.098	0.165	0.075	0.123
16.515	2-Decanone	0.044	0.036	0.041	0.033	0.044	0.057	0.037	0.037	0.043	0.118	0.035	0.04	0.041	0.068	0.031	0.038
16.695	Dodecane	0.305	0.19	0.084	0.079	0.345	0.353	0.073	0.078	0.247	0.224	0.091	0.097	0.326	0.47	0.057	0.125
16.890	Decanal	0.015	0.007	0.01	0.009	0.014	0.012	0.008	0.01	0.01	0.022	0.01	0.009	0.005	0.026	0.007	0.012
18.480	2-Decenal	0.074	0.054	0.09	0.081	0.081	0.082	0.088	0.054	0.071	0.192	0.084	0.07	0.058	0.09	0.05	0.076
18.610	Nonanoic acid	0.062	0.047	0.065	0.051	0.086	0.098	0.057	0.053	0.074	0.138	0.052	0.055	0.055	0.08	0.039	0.071
19.535	Tridecane	0.296	0.196	0.022	0.028	0.222	0.236	0.022	0.023	0.175	0.4	0.022	0.024	0.242	0.396	0.017	0.024
21.270	2-Undecenal	0.067	0.052	0.108	0.089	0.073	0.078	0.096	0.082	0.057	0.157	0.094	0.084	0.049	0.09	0.057	0.078
22.210	Tetradecane	0.122	0.067	0.056	0.054	0.121	0.145	0.05	0.05	0.102	0.267	0.066	0.059	0.146	0.213	0.035	0.061
22.460	Dodecanal	0.007	0.004	0.008	0.007	0.007	0.008	0.005	0.005	0.006	0.016	0.007	0.006	0.011	0.02	0.004	0.007
23.935	1-Dodecanol	0.114	0.068	0.089	0.079	0.077	0.108	0.073	0.072	0.085	0.27	0.089	0.079	0.125	0.258	0.052	0.088

RT.	Sample	ERBV 48h	ERBV 48h	ERBV 48h	ERBV 48h	ERBV 72h	ERBV 72h	ERBV 72h	EHV-1 24	EHV-1 24	EHV-1 24	EHV-1 24	EHV-1 48	EHV-1 48	EHV-1 48	EHV-1 48	EHV-1 72	EHV-1 72	EHV-1 72	EHV-1 72h 2
5.085	Butanoic acid	0	0	0	0.034	0	0	0	0.043	0.043	0.305	0.251	0.038	0.046	0	0	0.035	0	0.062	0
7.135	Pentanoic acid	0.046	0.047	0	0	0.078	0	0	0.054	0.051	0.551	0.344	0.045	0.052	0.043	0.059	0.042	0.041	0	0
7.325	Heptanal	0.03	0.022	0.024	0.034	0.04	0.019	0.013	0.037	0.037	0.317	0.221	0.028	0.021	0.022	0.027	0.029	0.021	0.023	0.031
9.425	Benzaldehyde	0	0	0	0	0.173	0.064	0.073	0	0	0.458	0.2	0	0	0	0	0	0	0	0
9.690	Dimethyl trisulfide	0.076	0.105	0	0	0.157	0.025	0.023	0.052	0.11	0	0	0.054	0.077	0.026	0	0.066	0.065	0.023	0.022
10.085	Hexanoic acid	0.063	0.081	0.091	0.086	0.119	0.07	0.068	0.082	0.098	1.247	0.729	0.079	0.084	0.081	0.137	0.065	0.065	0.131	0.078
10.665	Octanal	0.028	0.031	0.018	0.026	0.035	0.016	0.019	0.027	0.032	0.383	0.267	0.027	0.022	0.019	0.025	0.02	0.023	0.026	0.027
11.465	1-hexanol, 2-ethyl- /2-Propyl-1-pentanol	1.727	2.252	0.801	0.897	2.418	0.699	0.657	0.89	1.475	3.231	3.751	0.828	0.887	0.447	0.493	1.143	1.105	0.497	0.598
12.695	Acetophenone	1.238	1.525	0.687	0.845	1.92	0.685	0.59	0.604	0.801	2.585	2.389	0.537	0.628	0.463	0.443	0.931	0.943	0.663	0.661
12.820	1-Octanol	0.151	0.166	0.054	0.064	0.245	0.05	0.048	0.109	0.128	0.224	0.221	0.067	0.081	0.033	0.041	0.137	0.102	0.039	0.055
12.965	Heptanoic acid	0.062	0.057	0.09	0.1	0.096	0.058	0.062	0.083	0.085	1.131	0.767	0.057	0.08	0.093	0.115	0.058	0.057	0.115	0.077
13.850	Nonanal	0.11	0.104	0.04	0.064	0.113	0.044	0.035	0.061	0.069	0.762	0.583	0.061	0.052	0.053	0.076	0.055	0.058	0.048	0.076
15.940	Octanoic acid	0.07	0.102	0.112	0.118	0.115	0.095	0.094	0.091	0.094	1.472	0.918	0.081	0.088	0.132	0.159	0.071	0.081	0.169	0.109
16.515	2-Decanone	0.036	0.043	0.03	0.041	0.053	0.033	0.033	0.044	0.043	0.361	0.269	0.035	0.038	0.031	0.039	0.03	0.024	0.04	0.075
16.695	Dodecane	0.29	0.26	0.102	0.163	0.4	0.137	0.097	0.194	0.278	1.469	0.576	0.251	0.212	0.119	0.106	0.321	0.3	0.164	0.097
16.890	Decanal	0.01	0.015	0.011	0.016	0.017	0.01	0.008	0.01	0.014	0.168	0.081	0.012	0.009	0.01	0.01	0.012	0.007	0.011	0.013
18.480	2-Decenal	0.049	0.06	0.078	0.087	0.063	0.061	0.059	0.056	0.063	1.093	0.813	0.05	0.035	0.101	0.083	0.046	0.038	0.108	0.106
18.610	Nonanoic acid	0.038	0.048	0.059	0.057	0.057	0.054	0.053	0.046	0.051	0.872	0.523	0.068	0.045	0.092	0.121	0.034	0.048	0.118	0.066
19.535	Tridecane	0.34	0.138	0.025	0.038	0.306	0.029	0.021	0.13	0.254	0.377	0.129	0.201	0.141	0.031	0.019	0.135	0.17	0.042	0.03
21.270	2-Undecenal	0.045	0.056	0.076	0.092	0.057	0.064	0.055	0.044	0.055	1.256	0.882	0.044	0.047	0.107	0.106	0.047	0.042	0.116	0.114
22.210	Tetradecane	0.125	0.125	0.068	0.094	0.15	0.077	0.059	0.08	0.115	0.748	0.331	0.103	0.091	0.071	0.066	0.145	0.121	0.101	0.075
22.460	Dodecanal	0.007	0.009	0.011	0.012	0.012	0.009	0.007	0.006	0.007	0.077	0.032	0.006	0.007	0.006	0.005	0.006	0.006	0.008	0.009
23.935	1-Dodecanol	0.129	0.172	0.1	0.098	0.183	0.075	0.07	0.104	0.109	0.113	0.072	0.102	0.119	0.073	0.065	0.129	0.101	0.078	0.087

Table S1: Concentrations of VOCs obtained in each readout.
